# Assessment of Parents’ Oral Health Literacy and Its Association with Caries Experience of Their Preschool Children

**DOI:** 10.3390/children7080101

**Published:** 2020-08-18

**Authors:** Abdul Habeeb Adil, Sumaiya Zabin Eusufzai, Aimi Kamruddin, Wan Muhamad Amir Wan Ahmad, Nafij Bin Jamayet, Mohmed Isaqali Karobari, Mohammad Khursheed Alam

**Affiliations:** 1 Dental Public Health Unit, School of Dental Sciences, Universiti Sains Malaysia, Health Campus, Kubang Kerian, Kota Bharu 16150, Kelantan, Malaysia; drhabeebadil@gmail.com; 2 Pedodontics Unit, School of Dental Sciences, Universiti Sains Malaysia, Health Campus, Kubang Kerian, Kota Bharu 16150, Kelantan, Malaysia; draimi@usm.my; 3 Biostatistics Unit, School of Dental Sciences, Universiti Sains Malaysia, Health Campus, Kubang Kerian, Kota Bharu 16150, Kelantan, Malaysia; wmamir@usm.my; 4 Prosthodontics Unit, School of Dental Sciences, Universiti Sains Malaysia, Health Campus, Kubang Kerian, Kota Bharu 16150, Kelantan, Malaysia; dr.nafij@gmail.com; 5 Conservative Dentistry Unit, School of Dental Sciences, Universiti Sains Malaysia, Health Campus, Kubang Kerian, Kota Bharu 16150, Kelantan, Malaysia; dr.isaq@gmail.com; 6 Orthodontic Division, Preventive Dentistry Department, College of Dentistry, Jouf University, Sakaka, Aljouf 72345, Saudi Arabia; dralam@gmail.com

**Keywords:** caries, dmft, HUSM, oral health literacy and pedodontics

## Abstract

(1) Purpose: To assess the oral health literacy (OHL) of parents and its association with the caries experience of their preschool children attending the Hospital University Sains Malaysia (HUSM), Kota Bharu, Kelantan, Malaysia. (2) Materials and Methods: This is a descriptive cross-sectional study involving a systematic random sampling method, using a sample of 230 parent/preschool child dyads. Among 230 parents, 24 were males and 206 were females (mean age 31.43 ± 5.82); among 230 children, 92 were boys and 138 were girls (mean age 4.82 ± 1.04) attending the pedodontics clinic, HUSM, who participated and met the inclusion criteria. A structured, self-administered oral health literacy questionnaire including sociodemographic factors was used in this study. A child’s oral examination was performed to check the dmft (decayed, missing, filled teeth) status. Statistical analysis was done using descriptive and Spearman’s correlation analysis and multivariate regression analysis. (3) Results: The mean dmft score of children in relation to the OHL level of parents showed a significant difference (*p* < 0.00). The mean dmft score of children in relation to the OHL level of parents showed the following relationships: Inadequate (7.49 ± 4.10) followed by marginal (3.28 ± 2.67) and then adequate (0.55 ± 1.55). The incidence of caries amongst children in relation to parental employment was more associated with unemployed parents (6.11 ± 4.43) than with employed parents (2.79 ± 3.65). The caries experience amongst children in relation to education of their parents revealed a significant difference (*p* < 0.001), and the mean dmft score was high amongst preschool children with primary school qualified parents (10.7 ± 4.10) followed by high school (7.04 ± 3.68), vocational (5.81 ± 3.57), diploma (2.61 ± 2.81), and university (1.29 ± 2.27), respectively. The results revealed a valid significant difference (negative correlation, *r*_s_ = −0.753 **) between the OHL of parents with the dmft score of their preschool children. The age and gender of parents was not significantly associated with OHL, whereas ethnicity (positive correlation, *r*_s_ = 0.283 **), education (positive correlation, *r*_s_ = 0.865 **), and employment (negative correlation, *r*_s_ = −0.490 **) were found to be significant. Conclusion: We conclude that there is a significant association between the OHL of parents with the dmft score of their preschool children. The logistic regression showed that after adjustment for sociodemographic factors, parents’ gender (OR = 0.067, 95% CI: 0.012–0.360), parents’ employment status (OR = 3.247, 95% CI: 0.897–11.754), parents’ OHL score (OR = 0.042, 95% CI: 0.016–0.114), and child age (OR = 2.195, 95% CI: 1.249–3.857) were significantly associated with dental caries in children. Our study concluded that parents’ employment status, age, gender, OHL, and child’s age were significantly associated with the caries experience of their preschool children.

## 1. Introduction

Oral health literacy (OHL) is an important factor of oral health and general well-being of an individual [1]. The OHL has been subjected to little attention in dentistry until the last decade, when oral health practitioners and researchers have been increasingly interested in learning the association between oral health and health literacy, leading to development of the term OHL [2]. OHL is now emerging as a research field in dentistry [3]. The American Dental Association claims that individuals with low OHL constitute a hurdle to effective treatment, diagnosis, and prevention of dental diseases, and it has implemented an effective strategy to develop OHL among the population [4]. Moreover, researchers have recently stated that it is essential to improve individuals’ OHL to reduce oral health problems and produce better oral health outcomes in the community [2,5,6,7,8]. The Institute of Medicine committee on oral health and the American Dental Hygienist’s Association conducted a study including experts in health promotion, epidemiologists, physicians, nurses, dentists, and dental hygienists, showing that individuals’ literacy is an aspect that needs to be measured to evaluate the potential risk of general or oral health [9].

The definition of OHL proposed by the US Department of Health and Human Services/National Institutes of Dental and Craniofacial Research is “the manner in which people are able to access, manage and follow basic oral health information and resources that are essential to make effective oral health decisions” [10]. According to this definition, OHL includes reading, writing, speaking, listening, appropriate decision making, and numeracy skills, which are the ability to understand instructions on prescription drug bottles, appointment slips, medical education brochures, dental professionals’ directions, and consent forms. Data obtained from different studies have suggested that low OHL is associated with poorer oral health knowledge [11], and that patients with low OHL do not visit the dental clinic regularly or fail to show up for dental appointments [12]. Limited OHL may lead to low oral health-related quality of life [3]. Low OHL among individuals with oral diseases is also associated with more severe periodontal disease and worse self-reported oral health status [13,14]. Further, self-efficacy is suggested to influence the effects of literacy on oral health status [15].

The prevalence of dental caries is high with respect to diet and behaviour-related oral diseases in children. Dental caries is not dangerous but has a harmful effect on the quality of life, nutrition, eating ability, influence on self-esteem, and overall health of a child [16]. The early onset of caries and its infectious nature requires the utmost concern regarding preventive oral health care and primary treatment amongst children. Earlier studies have revealed that parents play an important role in prevention and management of caries amongst their children, and there is a direct association between oral hygiene behaviour of parents with oral health-related habits of their children [16,17]. The role of mothers is important in the first three years of a child’s life, as parents are still the primary caretakers of children’s oral health, even in their preschool period. Some factors such as age, parental education, employment, attitudes, behaviour, and knowledge are associated with improving healthy habits among themselves and in their children’s oral health secondarily [18].

OHL for parents of preschool children is very crucial because it may influence the oral health status of preschool children. In addition, knowing the OHL level of parents may be helpful for policymakers in terms of designing interventions and implementing oral health promotion strategies to reduce oral health problems of preschool children [19]. To the best of our knowledge, there has been no specific study to assess OHL in parents of preschool children of Kelantan state in Malaysia. Therefore, the objective of our study was to assess the association between OHL of parents and dmft (decayed, missing, filled teeth) status of their preschool children in Kelantan state in Malaysia.

## 2. Materials and Methods

This was a descriptive cross-sectional survey which included 230 parent/preschool child dyads. The study was conducted to determine the association of OHL level and sociodemographic characteristics among parents (mean age 31.43 ± 5.82), on caries experience of their preschool children (mean age 4.82 ± 1.04) attending the pedodontics clinic, Hospital University Sains Malaysia (HUSM). Ethical approval was procured from the Human Research and Ethics Committee (JEPeM) of University Sains Malaysia with number USM/JEPeM/19030211.

The sample size calculation was estimated by using the single mean formula $n={\left[\frac{z\times \sigma }{\Delta }\right]}^{2}$, where *n* = sample, *∆* = 1.2 precision, *z* = standard normal deviation to 95% confidence interval = 1.96, and *σ* = 15.64 reference from a previous study [20]. To determine the association between OHL level of parents and the dmft score of their preschool children, the sample size calculation was done using G* Power analysis [21]. The sample size was estimated to be 194. After adding a 20% non-response rate, a total of 232 parents and 232 preschool children were recruited for this study.

Inclusion criteria included healthy children aged 3–6 years and accompanied by at least one parent aged 22–51 years who was able to read and write in Bahasa Malaysian. Exclusion criteria included children with specific learning disabilities, caregivers other than parents, and parents who could not read and write in Bahasa Malaysian. Participants who were not willing to be part of the study were allowed to withdraw, as were patients without legal guardians. Data collection was done in the pedodontics screening clinic. The Bahasa Melayu version of the OHL-M questionnaire is a structured, validated, and self-administered questionnaire instrument to measure the OHL among the parents of preschool children. Training was provided to the research assistant based on the theoretical aspects of the instrument. The questionnaire was given to the parents, who were asked to complete the form in approximately 20 min. For parents who did not understand terminology or statements in the questionnaire due to their low educational level, an expert research assistant was appointed who was well versed on Bahasa Melayu to assist them.

### 2.1. Inter-Examiner and Intra-Examiner Reliability Assessment

A pilot test to assess the reliability among examiners was conducted on 23 (10%) randomly selected sample members prior to the clinical examination. The calibration of two clinical examiners for recording the dmft index was done according to protocols [22], and the oral examination of children was performed using common standards. The dmft data obtained by two examiners were compared for inter-examiner reliability using the SPSS software version 24.0 (kappa = 0.81). The same children were recalled after two weeks, the dmft status was recorded again by the examiner, and the obtained dmft data were compared with previous data recorded by the same examiner; the intra-examiner reliability kappa value obtained was 0.91. The results show a good reliability between the examiners. The children who were involved in this reliability test were not included in the main study.

### 2.2. Research Tool

The Bahasa Malaysia version of the Oral Health Literacy Instrument (OHLI-M) validated by Ramlay (2015) [19] was used to determine the level of OHL among participants. Prior to consent, the parent of the selected child is provided with a detailed explanation of the study to discuss concerns and procure acceptance for inclusion. The OHLI contains reading comprehension and numeracy sections. The reading comprehension section, which evaluates the capability of participants to read and understand written content, uses 38 passages for which participants must supply omitted words. The numeracy section, comprising 19 items, tests the capability of an individual to understand and interpret messages communicated in the form of numbers on prescription medicines, appointment cards, and post-operative instructions.

To calculate the score of the OHLI, each correctly answered item is given a score of 1. If the response is not correct or not known (unanswered), the item is scored as 0. The total score is the sum of all correct responses in both reading comprehension and numeracy. These scores are then multiplied by 1.31 (50/38) and 2.63 (50/19), respectively, to generate a weighted score ranging from 0 to 50 for each section. The total OHLI score ranges from 0 to 100 and is a replication of the sum of the scores from both the reading and numeracy sections, with higher values equalling greater OHL. The scoring of OHLI categorizes patients into three levels: Level I, inadequate (0–59), Level II, marginal (60–74), and Level III, adequate (75–100) [8].

The child’s dmft status was examined simultaneously by a qualified operator to provide details of the caries experience with the dmft score, by adding the total number of decayed or carious teeth (d), missed teeth (m), and the number of teeth with a filling(f) or crown. The dmft score ranges from 0 to 20 for a preschool child; dmft = 0 means the absence of dental decay, missing, and filled teeth, and dmft > 1 indicates the presence of decayed, missing, and filled tooth/teeth [20].

### 2.3. Statistical Analysis

Data were analysed using the SPSS v. 24.0. Data for numerical variables were expressed by the mean and the categorical variable was described in frequency and percentage (%). Associations between caries status with OHL and sociodemographic characteristics were analysed using Spearman’s correlation and regression analysis, which included caries status as the dependent variable and parents’ age, gender, employment, OHLM, and children’s age as independent variables. The Spearman’s rho correlation coefficient was computed to examine the associations among all variables. The Spearman’s correlation coefficient values can vary from −1.00 to +1.00. A correlation value of +1.00 indicates a perfect positive correlation, while a value of −1.00 represents a perfect negative correlation, and a value of 0.00 indicates no linear relationship between two variables.

## 3. Results

### Sociodemographic Profile

A total of 232 participants were involved initially, but the responses from two participants were excluded because they selected multiple answers for the same question. Hence, there were 230 total participant dyads (response rate = 99.13%): 230 parents (206 females and 24 males, mean age 31.43 ± 5.82), and 230 preschool children (138 females and 92 males, mean age 4.82 ± 1.04). Participants were divided into three groups depending upon ethnicity. Most of the parents held a basic university degree, and more parents were employed compared to unemployed. Table 1 shows the sociodemographic profile of the participants by age, gender, ethnicity, education, and employment.

Table 2 demonstrates the mean dmft score of preschool children in relation to the OHL level of parents, which showed a significant difference (*p* < 0.001). Caries status of preschool children was high among the inadequate (7.49 ± 4.10) level followed by marginal (3.28 ± 2.67) and adequate (0.55 ± 1.55) OHL levels of parents. The mean dmft score amongst children with unemployed (6.11 ± 4.43) parents was more when compared with that of employed parents (2.79 ± 3.65). A significant difference was found between the caries experience of children and parental employment (*p* < 0.001). The caries experience amongst children in relation to parental education revealed a significant difference (*p* < 0.001), and the mean dmft score was high amongst preschool children with primary school qualified parents (10.7 ± 4.10) followed by high school (7.04 ± 3.68), vocational (5.81 ± 3.57), diploma (2.61 ± 2.81), and university (1.29 ± 2.27), respectively.

Table 3 demonstrates the mean dmft score of preschool children according to their age and gender. The age (*p* < 0.001) and gender (*p* = 0.01) of preschool children was significant in relation to the dmft score. Mean dmft scores were higher in males (5.13 ± 4.59) than females (3.71 ± 4.12). A higher dmft was noted amongst children six years of age (6.85 ± 4.16), followed by five years (5.35 ± 4.14), four years (1.37 ± 2.60), and then three years (0.71 ± 1.78).

Table 4 tabulates the association of OHL and sociodemographic factors of parents with the dmft score of their preschool children. The results revealed a valid significant difference (negative correlation, *r*_s_ = −0.753 **) between OHL of parents with the dmft score of their preschool children. The age and gender of parents were not significantly associated with OHL, whereas ethnicity (positive correlation, *r*_s_ = 0.283 **), education (positive correlation, *r*_s_ = 0.865 **), and employment (negative correlation, *r*_s_ = −0.490 **) were found to be significant. There was a significant correlation between OHL of parents with their preschool children’s age (negative correlation, *r*_s_ = −0.710 **) and gender (positive correlation, *r*_s_ = 0.140 *). The association between dmft score of preschool children and the ethnicity, education, and employment of their parents was found to be significant (*r*_s_ = −0.270 **, *r*_s_ = −0.642 **, *r*_s_ = 0.403 **). Additionally, the age of the children revealed a significant association (*r*_s_ = 0.613 **). The associations between dmft score of preschool children and parents’ age and gender were found to be insignificant (*r*_s_ = 0.097 and *r*_s_ = −0.052).

Table 5 demonstrates the relation between the OHL level of parents across sociodemographic characteristics. Statistical analysis showed a significance difference in the OHL level of parents in relation to gender (0.03 *), ethnicity (0.00 *), education (0.00 *), and employment (0.00 *). No significant difference was seen in relation to the age of the parents (*p* = 0.92).

Logistic regression (Table 6) showed that after adjustment for sociodemographic factors, parent’s gender (OR = 0.067, 95% CI: 0.012–0.360), parents’ employment status (OR = 3.247, 95% CI: 0.897–11.754), parents’ OHL score (OR = 0.042, 95% CI: 0.016–0.114), and child’s age (OR = 2.195, 95% CI: 1.249–3.857) were significantly associated with dental caries in children.

**Figure 1:**
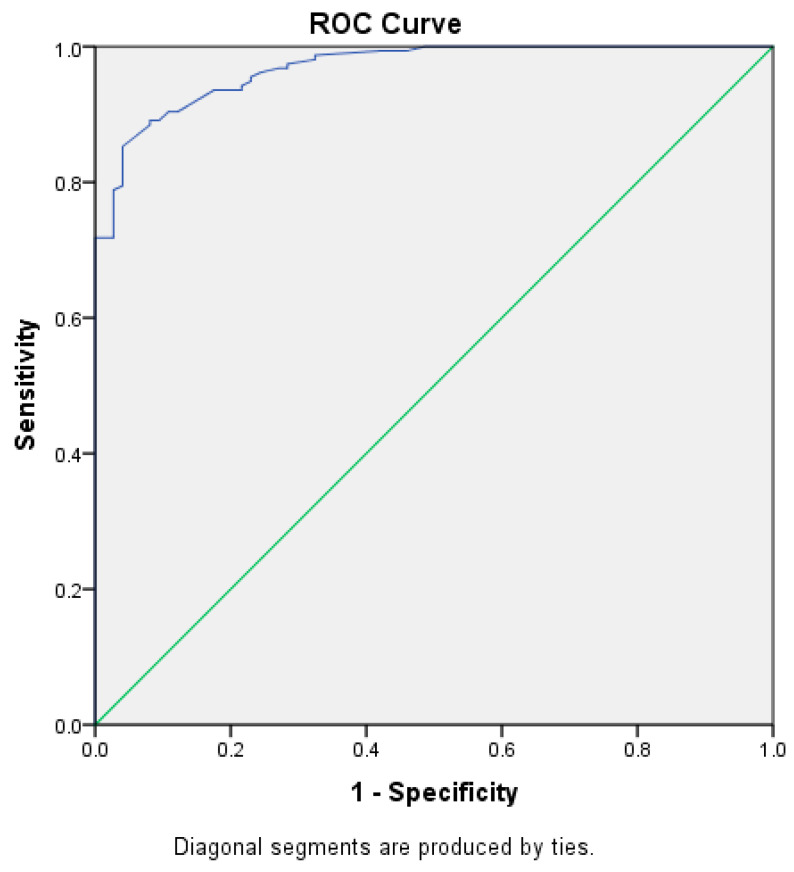
ROC curve. The area under the curve is 0.96. The model can accurately discriminate 96% of the cases.

Parents aged 34–43 had odds two times greater of having children with caries compared to those aged 22–33 and 44 and above 44. The odds of caries in children were about 85.3% lower for female parents compared to male parents. Unemployed parents had odds three times higher for having children with caries, compared to employed parents. Parents with high OHL scores had odds 95% lower of having children with caries, compared to parents with inadequate OHL scores. Child has increase in the age 1 year has 2 times the odds of caries present. The receiver operating characteristic (ROC) curve showed that the model can accurately discriminate 96% of the cases (Figure 1).

## 4. Discussion

OHL has become an important factor of concern in regard to oral health [23,24,25]. The literature has shown high caries risk among children in association with low parental OHL [1,6,26,27,28]. A study revealed that parents with higher OHL showed a negative correlation with their children’s caries experience, with the authors concluding that parents with adequate OHL play an important role in prevention and management of children’s caries experience [29].

This study’s findings about the OHL of parents—inadequate level 43.9%, marginal level 24.8%, and adequate level 31.3%—are in agreement with a study conducted among the Brazilian population by Firmino et al. [30], where the OHL level of the participants was found to be inadequate (37%), and also among the Iranian population, where more than half of the participants had an inadequate and marginal (55.7%) OHL level [31]. Furthermore, in agreement with our study, the OHL level of participants was found to be inadequate or poor (49%) among the Bangladeshi population [32] and among the Indian population, where 35% of participants had an inadequate OHL level [33]. An inadequate level of OHL has been found in various studies and is consistent, possibly because of low education levels, people living in rural areas, ethnic minorities, and low socioeconomic status among the studied populations [30,31,32,33]. Our study results revealed a higher inadequate level of OHL among parents with primary school qualifications and among unemployed parents, which shows that education and employment are important factors for good OHL levels. In contrast with our findings, a study conducted by Fabillah et al. [34], using the same tool to assess the OHL among carers of special needs children in Kuala Terengganu Malaysia, where the majority of the participants had an adequate level of OHL, revealed that there is a need to develop more suitable intervention programs for the carers of children with special needs to attain OHL [34].

The OHL level of parents in relation to the mean dmft score of their preschool children showed a significant difference (*p* = 0.00 *). The dmft score of children was more in relation to inadequate OHL of parents (7.49 ± 4.10), followed by marginal (3.28 ± 2.67), and then adequate (0.55 ± 1.55) OHL levels. This agrees with studies conducted among preschool children of India (*p* < 0.00 *) [35], Iran (*p* = 0.005 *) [7,16], Hong Kong (*p* < 0.001 *) [29], and Brazil (*p* < 0.001 *) [36].

Parents with inadequate OHL must be provided with guidance on oral health education through promotion and intervention programs. The OHL of parents can be improved with good oral health promotion, which should be carried out at the early preschool level of their children. The parents should educate their children at home, which has an influence on the behaviour and attitude of children, just as how a child learns language [33,37]. The government of Malaysia has been implementing various health promotion and health education programs in the oral health field, such as the National Oral Health Plan 2011–2020, which aims to decrease the prevalence of dental caries to less than 50% by the year 2020 among the Malaysian population [38]. It is important to enhance the OHL level of the parents by health promotion strategies that involve the mass media, television advertising, the Internet, pamphlets, and brochures, etc. Intervention programs should also include education and health education advice through interviews, preventive care, and clinical examinations. This would improve parents’ knowledge, habits, behaviour, attitudes, and practices, which in turn would improve the oral health status of their preschool children.

Childhood oral health depends on the awareness of their parents, as the OHL of parents affects the oral health-associated lifestyles of children, which are developed during the initial stages of childhood and continue in later stages [39,40,41]. The prevalence of caries among the Malaysian adult population was reported as 88.9% according to the National Oral Health Survey of Adults in Malaysia [42]. According to the Malaysian ministry of health, the prevalence of dental caries among preschool children was reported to be 71.3% [43].

The dmft score amongst preschool children in the current study was shown to be 68% carious, and found to be more amongst six-year-old children, followed by children aged five, four, and then three years, in agreement with other studies conducted amongst Malaysian children, which revealed 70–90% [2], 80.6% [44], and 74.6% [45], respectively. The mean dmft score of preschool children was 4.37 where 32.2% were caries free out of 230 preschool children, in agreement with a study conducted amongst Malaysian children showing 6.24 [46], 5.27 [45], and Hong Kong children 5.2 [19]. This is in contrast to a study conducted among Iranian children, where the mean dmft score was 8.2 [16]. The mean dmft score with respect to the age of preschool children revealed a significant difference (*p* = 0.00 *). This agrees with studies conducted among preschool children of China, which showed that the prevalence of caries increased with age [47], and among Italian preschool children, where the severity of caries also increased with age—three years (15%), four years (24%), and five years (31%) [48]. Furthermore, among Malaysian preschool children (*p* = 0.03 *) [49] and among Sudanese preschool children which concludes higher dmft scores as the age increases [50]. Our study results revealed a statistical difference between mean dmft score and gender of the preschool children (0.01 *), where the mean dmft score was higher among male children (5.13 ± 4.59) than among female children (3.71 ± 4.12). Similar results were found in studies conducted among Brazilian preschool children, where the mean dmft score was higher among males (1.65 ± 1.31) compared to females (1.15 ± 2.03) [51], Saudi preschool children (males 5.3 ± 3.7; females 5.1 ± 4.3) [28] and Malaysian preschool children (males 5.41 ± 4.94; females 5.15 ± 5.46) [45].

Oral health plays a significant role in the general well-being of individuals. Meanwhile, good OHL can have a positive influence on oral health. Certainly the acceptance of good oral health habits in childhood frequently takes place in the presence of their parents, mainly with mothers.

The OHL of mothers with good habits such as brushing, proper diet, and choice of food are directly related with their children’s oral health status. Therefore, improving the OHL of parents might result in decreased caries experience in their children. Studies have shown that many factors, such as low OHL, genetics, bad oral health behaviour, and improper care of oral health in parents, are directly associated with the oral health status of their children [18,52]. Improved OHL in parents is related to their oral health behaviour, hygiene, attitudes, knowledge, and practice, which in turn influences their children in achieving a better oral health status. In rural areas, unhealthy and sugary foods and beverages are common. Additionally, limited access to dental health services and oral health promotion programs may contribute to poor children’s oral health in rural communities [16].

Earlier studies have shown an association between the OHL of parents and clinical outcomes; however, research to determine the association between parental OHL and caries experience of children is still required [7,46].

Our results revealed that there is a significant association between the OHL literacy of parents and the dmft status of their preschool children. The Spearman’s correlation coefficient showed a negative correlation *r*_s_ = −0.753 ** in relation to the dmft status of preschool children. This is in agreement with studies conducted amongst Malaysian and Hong Kong children, which showed negative correlations of −0.326* [46] and −0.278 ** [19], respectively. Further, it also agrees with studies conducted among Brazilian children (*p* < 0.001 *) [53] and among American children (*p* > 0.05 *) [54]. This negative association was predictable because when the level of OHL in parents increases, the dmft status of children will improve and show lower caries experience. In contrast, a study conducted amongst Somali immigrant children in America showed that parents’ OHL level was not significantly associated (*p* = 0.67) with the dmft status of their children [55]. The literature has revealed a significant association between lower parental OHL and high prevalence of caries amongst their children [6,53,56].

This study showed a significant association between the education of parents and the dmft score of their preschool children (*r*_s_ = −0.642 **). This is in agreement with studies conducted amongst the Iranian population, which showed a negative relation between high parental education level and low dmft status of their children (*p* = 0.05 *) with mean dmft of 5.84 [57]; the American population, which showed a significant association between high education level of parents and less caries experience in their children (*p* = 0.0001 *) [58]; Hong Kong parents and their children, which revealed a significant association (*p* ˂ 0.001); and amongst the Malaysian population, which showed a significant association (*p* ˂ 0.05) [29,46]. Furthermore, the association of parents’ employment in relation to the dmft score of their children showed a significant difference (*r*_s_ = −0.403 **), which is in agreement with studies conducted amongst the Malaysian population (*p* ˂ 0.05) [44,46] and the Hong Kong population (*p* ˂ 0.001) [29]. The literature has revealed that the socioeconomic status of parents is significantly associated with the dmft score of their preschool children [46,59].

The OHL level of parents showed a significant difference in relation with their ethnicity (*p* = 0.00 *) and education (*p* = 0.00 *). The OHL level was higher among the Malay (91.7%) population compared to Chinese (4.3%) or Indian (3.9%). This is in agreement with studies conducted by Baskaradoss [60], where the OHL level of participants was significantly associated with race (0.046 *) and educational level (0.004 *); among the Malaysian population, where 62.5% of parents had a high OHL level [46]; in the American population (*p* = 0.02 *), where higher educational level was associated with improved OHL [61]; and among Iranians (*p* = 0.001 *), which revealed that participants with less education had a lower level of OHL [31]. In contrast, no significant difference was noted in the OHL level of parents and their ethnicity in a study conducted amongst Malaysians [42]. A good educational background has an important influence on the OHL level of parents, because well-educated individuals have access to oral health care materials [62,63]. Lower educational level leads to inadequate behaviour and poor oral health results [64]. This study showed no significant difference in relation to the OHL level of parents with their age (*p* = 0.92), which is in agreement with a study done by Naghibi Sistani et al. [65] among the Iranian population. The OHL level was found to be higher among the younger population compared to the older age group, which could be due to exposure to numerous information resources and a higher capability of absorbing and practicing this information among the younger generation compared to older age groups [62]. The employment status (0.00 *) of the parents showed a significant relation with the OHL level. This agrees with a study conducted by Noor et al. [42], wherein the authors revealed that 20% of the Malaysian population had significantly higher OHL scores when compared with different income categories. The literature has revealed that the employment status of the parents plays a significant role in improved OHL [64,66]. Individuals with a good economic status have better access to oral health education and promotion sources, as well as more convenient access to dental health providers, which in turn improves their OHL.

Our study findings revealed a significant difference between gender (0.03 *) of the parents and OHL. The OHL among females (89.6%) was higher compared to males (10.4%). A similar outcome was found in a study conducted in India. [67], wherein female (89.6%) participants had a high level of OHL compared to males (7.7%), and also a study conducted among the Indian population, where the OHL score (*p* = 0.032 *) was higher in females compared to males [33]. The OHL level was found to be significantly higher among females (−0.35 *) than among males (0.00 *), possibly due to more use of dental health services by females [11]. The study results indicate that female participants had better OHL skills, in following oral health instructions and in making suitable decisions for an improved oral health status, compared to male participants.

The education and employment of parents has a direct influence on their OHL level. It is essential to improve the overall educational level of people in the community, which will enhance the OHL level of parents and subsequently lead to better health outcomes and better oral health status among their children. Our study covered parents with different education levels (primary, secondary, vocational, diploma, and university level of education). Parents and their preschool children attended the research and completed the survey, a good response has been shown by the participants. Our research has a community health association as well. This offers a framework for designing and implementing oral health programs which enhance the OHL, skills, and attitude of parents. It also provides ideas to enhance interactions between health care providers and parents, which could improve the oral health status among children [26].

The logistic regression model (Table 6) showed that after adjustment for sociodemographic factors, parents’ employment status was significantly associated with the dental caries status of preschool children. This particular finding has been supported by pertinent study performed in Japan [68]. This may indicate that financial struggles create inequality in access to oral health education, information, and interventions that are required for parents to improve the oral health status of their preschool children [29]. It is obvious that employed parents can afford to seek oral care and ensure basic oral hygiene practices, for example, toothbrushes, fluoridated toothpaste, etc., and they can improve behaviour towards oral health care by sharing knowledge with friends and colleagues [42]. It is obvious that the sociodemographic status, along with the level of OHL, are substantial instigating factors to increase access to oral health care. [29]

In this study, we observed the influence of sociodemographic disparities in clinical oral health status. Parental employment status was found to be associated with dental caries status. These findings confirm previous studies that have shown sociodemographic divergence in different parts of the world [16,26,29,68,69]. Although from this cross-sectional study design it was hard to show causality between factors that contribute to the oral health status of preschool children, the analysis of multivariate models leads us to reach two conclusions. Parents’ OHL score is a direct determinant which interacts with other direct determinants and is affected by intermediate determinants such as the child’s age and the parents’ employment status. Parents’ age, gender, and age of children were also associated with the presence of caries (*p* < 0.05), which is similar to a study performed in Hong Kong [29]. Prolonged exposure to caries risk factors and a gradual reduction of parents’ control over their children’s nutrition, as well as their hygienic behaviour as they mature might be responsible for this [16].

### 4.1. Strength of the Study

To the best of our knowledge, this was the first study performed in the Kelantan district assessing oral health literacy and its association with the caries status. It is of sound design and of clear practical and clinical interest. The obtained data will help the governmental sector to develop health promotion strategies and interventional programs, which can further help in the prevention of dental caries. Additionally, the questionnaire used in this study (OHLI-M) is an instrument that measures the ability to read and understand medical instructions and health care information presented in prose passages, as well as passages containing numerical information (e.g., prescription bottle labels and appointment slips), and it was validated in the Malay language.

### 4.2. Limitation of the Study

The present study was limited to a cross-sectional design, and all the subjects were among the patients referred to the pedodontics clinic, USM. Consequently, our study findings may not represent the population as a whole, and they should be interpreted cautiously.

Among the functional domains of oral health literacy, the OHLI-M can assess only reading comprehension and numeracy, but not listening and decision making. Numeracy skills do not necessarily indicate that the person understands the meaning of the words. Since written and spoken Kelantanese are different from the Bahasa Malay language, further research is needed to extrapolate oral health literacy levels in the Kelantanese dialect. However, to overcome this problem, a research assistant having a command of the Kelantanese dialect was available during data collection. As we are aware that preschool children themselves do not have access to oral health care, parents therefore are required to provide this access, by their own initiative. The literature has suggested that parents with lower socioeconomic status might be reluctant to allow their preschool children to access dental treatment, probably because of cost concerns. They may also have a misconception that dental treatment for their child’s dental problems is less important because the primary teeth will be shed off. In order to increase oral health access for preschool children, more steps should be taken to promote oral health care to the public, specifically for parents with lower socioeconomic status. Under these circumstances, oral health education materials should be designed for parents with low literacy levels so that they can read and easily understand and grasp the basic oral health concepts and prevention strategies for oral diseases. Strong emphasis on prevention strategies, for example, non-cariogenic foods, basic oral health care, etc., should be put in place to limit the financial burden of dental treatment, focusing on this group of parents. Furthermore, another limitation of this study is that it assessed only dental caries and not also the gingivitis/periodontal condition of the child.

This study’s results will help policymakers design different educational, interventional, and promotion programs to improve OHL among the population, which in turn can lead to a reduction in the prevalence of dental caries in children. The intervention should be done through schools and other various means, such as television, the Internet, newspapers, radio, hospital pamphlets, and posters, all of which could enhance the OHL of the general population. Oral health education should be implemented in the school curriculum to develop better OHL skills. A home visiting program can be introduced to improve OHL among families for the prevention of oral diseases. Dental professionals should use simple language to educate patients to increase their OHL level by using suitable communication techniques (verbal and visual aids), encourage parents to ask questions, and make sure they understand and follow the instructions properly. Non-dental professionals such as nursing practitioners can also contribute to the prevention of early dental caries among children by educating parents and improving their OHL.

## 5. Conclusions

Our study concludes that the caries experience among preschool children attending HUSM Kelantan was found to be high, and the OHL of parents was significantly associated with the caries experience of their preschool children. Further, education and employment were also significantly associated with the caries experience of parents’ preschool children. The OHL of parents has a benefit in improving children’s oral health status. Since this study was done only on children who visited HUSM for dental treatment, it is suggested that more studies should be carried out in different parts of the country for a large sample size to evaluate the OHL of parents. This could help in developing health promotion strategies and different interventional programs by the public health sector that in turn could reduce the prevalence of dental caries among the children.

## Figures and Tables

**Table 1:** Sociodemographic profile of the parents and their preschool children.

Demographic Variables	*n* (%)
**Age of the Parent**	
22–33 years	136 (59.1)
34–43 years	79 (34.4)
44 years and above	15 (6.5)
**Gender of the Parent**	
Female	206 (89.6)
Male	24 (10.4)
**Ethnicity**	
Malay	211 (91.7)
Chinese	9 (4.3)
Indian	10 (3.9)
**Education**	
Primary School	19 (8.3)
High School	52 (22.6)
Vocational	32 (13.9)
Diploma	42 (18.3)
University	85 (37)
**Employment**	
Employed	129 (56.1)
Unemployed	101 (43.9)
**Age of the Child**	
3 years	32 (13.9)
4 years	54 (23.5)
5 years	67 (29.1)
6 years	77 (33.5)
**Gender of the Child**	
Male	92 (40)
Female	138 (60)

**Table 2:** Mean decayed, missing, filled teeth (dmft) score of preschool children in relation to the oral health literacy (OHL) level of parents.

Variables	Caries Status		Dmft	*p* Value
	Caries Present	Caries Absent	Mean ± SD	
**OHL level**				
Inadequate	101	0	7.49 ± 4.10	<0.001 *
Marginal	44	13	3.28 ± 2.67	
Adequate	11	61	0.55 ± 1.55	
**Employment**				
Employed	66	63	2.79 ± 3.65	<0.001 *
Unemployed	90	11	6.11 ± 4.43	
**Education**				
Primary school	19	0	10.7 ± 4.10	<0.001 *
Secondary school	52	0	7.04 ± 3.68	
Vocational	32	4	5.81 ± 3.57	
Diploma	27	11	2.61 ± 2.81	
University	26	59	1.29 ± 2.27	

* Significant value *p* < 0.05.

**Table 3:** Mean dmft score of preschool children, by age and gender.

Variables	Caries Free	Caries Present	Decayed	Missing	Filled	Dmft Mean ± SD	*p* Value
**Age**							
3 years	26	06	0.65 ± 1.65	0.00 ± 0.00	0.62 ± 0.24	0.71 ± 1.78	<0.001 *
4 years	34	20	1.09 ± 2.07	0.01 ± 0.13	0.25 ± 0.70	1.37 ± 2.60	
5 years	11	56	4.25 ± 3.00	0.10 ± 0.35	1.00 ± 1.30	5.35 ± 4.14	
6 years	03	74	5.50 ± 3.28	0.22 ± 0.47	1.12 ± 1.15	6.85 ± 4.16	
Total	74	156	3.43 ± 3.40	0.10 ± 0.35	0.73 ± 1.11	4.27 ± 4.36	
**Gender**							
Male	24	68	4.04 ± 3.45	0.17 ± 0.45	0.91 ± 1.29	5.13 ± 4.59	0.01 *
Female	50	88	3.02 ± 3.31	0.65 ± 0.24	0.62 ± 0.73	3.71 ± 4.12	
Total	74	156	3.43 ± 3.40	0.10 ± 0.35	0.73 ± 1.11	4.27 ± 4.36	

* Significant value *p* < 0.05.

**Table 4:** Association of OHL and sociodemographic factors.

Sl No.	Variables	1	2	3	4	5	6	7	8	9
1	Dmft	1.000								
2	OHL	−0.753 **	1.000							
3	Age of parents	0.097	−0.036	1.000						
4	Gender of parents	−0.052	−0.126	−0.151 *	1.000					
5	Ethnicity	−0.270 **	0.283 **	−0.058	0.053	1.000				
6	Education	−0.642 **	0.865 **	0.008	−0.175 **	0.195 **	1.000			
7	Employment	0.403 **	−0.490 **	−0.025	0.245 **	−0.138 *	−0.537 **	1.000		
8	Age of child	0.613 **	−0.710 **	0.009	0.097	−0.088	−0.652 **	0.468 **	1.000	
9	Gender of child	−0.106	0.140 *	−0.152 *	0.070	0.056	0.150 *	−0.029	−0.121	1.000

** Correlation is significant at the 0.01 level (2-tailed); * correlation is significant at the 0.05 level (2-tailed).

**Table 5:** Demonstrates the distribution of parents with their level of OHL across sociodemographic characteristics.

Variable	OHL Level			χ^2^ (df)	*p* Value
	Adequate	Marginal	Inadequate		
**Age**					
22–33	43(18.7%)	36(15.7%)	57(24.8%)	0.931(4)	0.92
34–43	25(7.4%)	17(7.4%)	37(16.1%)		
44 and above	4(1.7%)	4(1.7%)	7(3%)		
**Gender**					
Female	64(27.8%)	45(19.6%)	97(42.2%)	11.441(2)	0.03 *
Male	8(3.5%)	12(5.2%)	4(1.7%)		
**Ethnicity**					
Malay	58(25.2%)	53(23%)	100(43.5%)	21.097(4)	0.000 *
Chinese	6(2.6%)	3(1.3%)	1(0.4%)		
Indian	8(3.5%)	1(0.4%)	0(0%)		
**Education**					
Primary	0(0%)	0(0%)	19(8.3%)	231.502(8)	0.000 *
High school	0(0%)	1(0.4%)	51(22.2%)		
Vocational	0(0%)	4(1.7%)	28(12.2%)		
Diploma	12(5.2%)	27(11.7%)	3(1.3%)		
University	60(26.1%)	25(10.9%)	0(0%)		
**Employment**					
Employed	59(25.7%)	42(18.3%)	28(12.2%)	59.704(2)	0.000 *
Unemployed	13(5.7%)	15(6.5%)	73(31.7%)		

* Significant value *p* < 0.05.

**Table 6:** Factors associated with dental caries: Regression analysis.

	Variable	Adjusted Model	*p* Value	Unadjusted Model	*p* Value
		OR (95% CI, Lower-Upper)		OR (95% CI, Lower-Upper)	
Caries status	Parents age				
	22–33 years	1		1	
	34–43 years	2.406 (0.777–7.449)	0.128	1.556 (0.845–2.862)	0.155
	44 above	1.389 (0.161–27.399)	0.563	1.549 (0.468–5.126)	0.474
	Gender	0.067 (0.012–0.360)	0.002	0.676 (0.257–1.782)	0.429
	Employment	3.247 (0.897–11.754)	0.073	7.810 (3.821–15.963)	<0.001
	OHLM	0.042 (0.016–0.114)	<0.001	0.39 (0.018–0.085)	<0.001
	Childs age	2.195 (1.249–3.857)	0.006	5.455 (3.537–8.414)	<0.001

Significant value < 0.05. Caries status model: Adjusted model: Dependent variable (dmft); Independent variable: Parents Age, Parents Gender, Parents Employment status, Parents OHLM score; Child’s Age. Unadjusted model: Dependent variable (dmft); Independent variable: Parents Age, Parents Gender, Parents Employment status, Parents OHLM score; Child’s Age.
